# P-459. Cytokine Profiles as Biomarkers of Bloodstream Infections in Children with Febrile Neutropenia after Hematopoietic Cell Transplant

**DOI:** 10.1093/ofid/ofaf695.674

**Published:** 2026-01-11

**Authors:** Sandra Castejon-Ramirez, Gabriela Maron, Marie Wehenkel, Sherri Surman, Victoria Henderson, Randall Hayden, Octavio Ramilo, Asuncion Mejias

**Affiliations:** St. Jude Children's Research Hospital, Memphis, TN; St. Jude Children's Research Hospital, Memphis, TN; St. Jude Children's Research Hospital, Memphis, TN; St. Jude Children's Research Hospital, Memphis, TN; St. Jude Children's Research Hospital, Memphis, TN; St. Jude Children's Research Hospital, Memphis, TN; St. Jude Children's Research Hospital, Memphis, TN; St Jude Children's Research Hospital, Memphis, TN

## Abstract

**Background:**

Bloodstream infections (BSI) before neutrophil recovery are a significant cause of morbidity and mortality after hematopoietic cell transplant (HCT) in children. Febrile neutropenia (FN) occurs in 80% of children post-HCT but BSI are only identified in 30% by routine blood cultures. Cytokines have been studied as potential biomarkers for BSI in immunocompromised adults with FN, but data post-HCT in pediatric patients is limited. This study aims to evaluate the role of serum cytokine profiles as predictive markers of BSI in children and adolescents with FN post-HCT.

**Figure 1.**
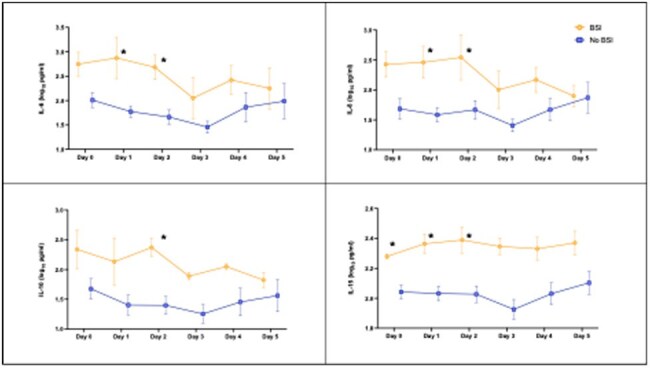
Temporal trends of IL-6, IL-8, IL-10, and IL-15 in BSI vs non-BSI post-HCT. Data expressed as mean log10 concentrations +/- SEM.

**Methods:**

We conducted a retrospective study from April to June 2024 and measured plasma cytokine concentrations in sequential blood samples initially collected per standard of care in pediatric -HCT patients, starting at day 0 of FN and until neutrophil engraftment for a median of 11 days (IQR: 10.3, 12.8). Samples were analyzed using a multiplex immunoassay (MILLIPLEX®) that included Th1, Th2, Th17, interferon (IFN) and inflammation related cytokines. Cytokine profiles were compared between children who developed a BSI vs those who did not.

**Results:**

We analyzed 15 episodes of FN post-HCT from 14 patients, of which 4 (26.7%) had a BSI with blood cultures revealing *K. pneumoniae, S. mitis, K. oxytoca and C. parapsilosis*. Median age was 9.4 years (IQR: 5.2, 12.4) and 5 (35.7%) were female. Underlying diagnoses included acute leukemia in 9 children and neuroblastoma in 5. Nine patients (64.3%) received an allogeneic HCT and 5 (35.7%) an autologous HCT. Median time from HCT to FN was 3 days (IQR: 0, 4.5) and samples were collected at a median of 10 hours (IQR: 4.2, 16.4) from FN onset. Plasma concentrations of IL-6, IL-8, IL-10, and IL-15 were higher in children with BSI. These differences first reached statistical significance on day (D) 0 for IL-15; on D1 for IL-6, IL-8, IL-15; and on D2 for the 4 cytokines (Fig 1). No differences were observed in Th17 and IFN related cytokines.

**Conclusion:**

Elevated IL-6, IL-8, IL-10, and IL-15 concentrations were associated with BSI post-HCT. These initial results emphasize the value of cytokine profiles for the early diagnosis of BSI after HCT in children and adolescents.

**Disclosures:**

Gabriela Maron, MD, MS, SymBio Pharamaceuticals: Advisor/Consultant|SymBio Pharamaceuticals: Grant/Research Support Randall Hayden, MD, Abbott: Board Member|Abbott: Serving on the advisory board|Cepheid: Board Member|Cepheid: Serving on the advisory board|Roche Diagnostics: Advisor/Consultant|Roche Diagnostics: Board Member|Roche Diagnostics: Serving on the advisory board Octavio Ramilo, MD, Merck: Advisor/Consultant|Merck: Grant/Research Support|Merck: Honoraria|Moderna: Advisor/Consultant|Pfizer: Advisor/Consultant|Pfizer: Honoraria|Sanofi: Advisor/Consultant Asuncion Mejias, MD, PhD, MsCS, Enanta: Advisor/Consultant|Merck: Grant/Research Support|Moderna: Advisor/Consultant|Pfizer: Advisor/Consultant|Sanofi-Pasteur: Advisor/Consultant

